# Short-term exposure to air pollution and hospital admission for pneumonia: a systematic review and meta-analysis

**DOI:** 10.1186/s12940-020-00687-7

**Published:** 2021-01-07

**Authors:** Jeong Yee, Young Ah Cho, Hee Jeong Yoo, Hyunseo Yun, Hye Sun Gwak

**Affiliations:** 1 College of Pharmacy and Graduate School of Pharmaceutical Sciences, Ewha Womans University, 52 Ewhayeodae-gil, Seodaemun-gu, Seoul, 03760 Republic of Korea; 2 College of Pharmacy, Gyeongsang National University, Jinju, Gyeongnam 52828 Republic of Korea; 3 Mokhwa Convalescent Hospital, Jinju, Gyeongnam 52828 Republic of Korea; 4 Department of Pharmacy, National Medical Center, Seoul, 04564 Republic of Korea; 5 Graduate School of Clinical Biohealth, Ewha Womans University, Seoul, 03760 Republic of Korea

**Keywords:** Air pollutants, Particulate matter, Pneumonia, Systematic-review, Meta-analysis

## Abstract

**Background:**

Air pollution is a major issue that poses a health threat worldwide. Although several studies investigated the adverse effects of air pollution on various diseases, few have directly demonstrated the effects on pneumonia. Therefore, we performed a systematic review and meta-analysis on the associations between short-term exposure of air pollutants and hospital admission or emergency room (ER) visit for pneumonia.

**Methods:**

A literature search was performed using PubMed, Embase, and Web of Science up to April 10, 2020. Pooled estimates were calculated as % increase with 95% confidence intervals using a random-effects model. A sensitivity analysis using the leave-one-out method and subgroup analysis by region were performed.

**Results:**

A total of 21 studies were included in the analysis. Every 10 μg/m^3^ increment in PM_2.5_ and PM_10_ resulted in a 1.0% (95% CI: 0.5–1.5) and 0.4% (95% CI: 0.2–0.6) increase in hospital admission or ER visit for pneumonia, respectively. Every 1 ppm increase of CO and 10 ppb increase of NO_2_, SO_2_, and O_3_ was associated with 4.2% (95% CI: 0.6–7.9), 3.2% (95% CI: 1.3–5.1), 2.4% (95% CI: − 2.0-7.1), and 0.4% (95% CI: 0–0.8) increase in pneumonia-specific hospital admission or ER visit, respectively. Except for CO, the sensitivity analyses yielded similar results, demonstrating the robustness of the results. In a subgroup analysis by region, PM_2.5_ increased hospital admission or ER visit for pneumonia in East Asia but not in North America.

**Conclusion:**

By combining the inconsistent findings of several studies, this study revealed the associations between short-term exposure of air pollutants and pneumonia-specific hospital admission or ER visit, especially for PM and NO_2_. Based on the results, stricter intervention policies regarding air pollution and programs for protecting human respiratory health should be implemented.

**Supplementary Information:**

The online version contains supplementary material available at 10.1186/s12940-020-00687-7.

## Background

Pneumonia is a common but potentially life-threatening disease with a high incidence around the world [1]. It is considered a leading infectious cause of hospitalization and death with rising health care costs [2]. The annual incidence of pneumonia is around 2.5 cases per 1000 adults with almost 20% requiring intensive care and 2% deaths. In terms of management, the cost of inpatient care is 25 times that of outpatient care [3].

Air pollution still poses a major health threat worldwide. It has been reported that over 90% of the world’s population live in areas where the air pollution level exceeds the World Health Organization (WHO) guideline limits [4]. Air pollution has raised serious concerns regarding environment and public health [5]. According to WHO, ambient air pollution accounted for 4.2 million deaths in 2016, which represented 7.6% of all deaths worldwide [4]. The adverse effects of air pollution have been investigated on various diseases [6–8].

The air pollution can increase the onset risk of pneumonia, including both hospitalization [9, 10] and outpatient visit [11]. In addition, particulate matter (PM) can worsen the prognosis of pneumonia patients; according to Chen et al. [12], it was associated with increased risk of invasive respiratory and/or vasopressor support and in-hospital mortality.

Although several studies systemically reviewed the effects of particulate matter (PM) on hospital admissions for respiratory diseases, such as asthma and chronic obstructive pulmonary disease (COPD) [13, 14], few have directly investigated the effects of air pollutants on pneumonia-specific hospital admission or emergency room (ER) visit. To summarize the existing evidence and provide a quantitative answer to the above concerns, we performed a systematic review and meta-analysis for the association between short-term exposure of air pollutants and hospital admission or ER visit for pneumonia.

## Methods

### Literature search strategy

The literature search was performed using PubMed, Embase, and Web of Science for studies on the association between short-term exposure to air pollution and hospital admission or ER visit for pneumonia up to April 10, 2020. The search included keywords related to air pollution (PM_2.5_, PM_10_, SO_2_, NO_2_, CO, and O_3_) and hospital admission or ER visit for pneumonia. The search strategy is detailed in Supplementary Table 1, Additional file 1. After removing duplicates, two researchers independently screened the titles and abstracts of all records to identify potentially eligible studies. Then, a full-text review was performed to determine the final inclusion according to eligibility criteria. In cases of disagreement, a consensus was reached by discussion.

### Inclusion and exclusion criteria

Studies were included if they: (1) were original studies published in peer-reviewed journals, (2) investigated the short-term effects (defined as those occurring up to 5 days prior to the hospital admission or ER visit) of air pollutants on hospital admission or ER visit for pneumonia, (3) provided sufficient information to calculate regression estimates and 95% confidence intervals (CIs), (4) used time-series or case-crossover study design, and (5) were published in English. Exclusion criteria were: (1) reviews, commentaries, or editorials; (2) in vitro or in vivo studies; (3) studies on children only; (4) studies under special conditions (e.g., high and low temperature) without overall estimates; or (5) studies on combined outcomes with other respiratory diseases. If there were overlapping data, only the most recent and comprehensive data were included in the meta-analysis.

### Data extraction

Data were extracted independently by two researchers and discrepancies were resolved by consensus. The following information was extracted from each study: name of the first author, publication year, study setting, study design, number of cases, percentages of male and elderly patients, air pollutants studied, outcome level, and study results. If there were multiple lag estimates for the same exposure, only one estimate was selected to prevent over-representation of a single study in the meta-analysis. For multiple lag estimates, a priori lag selection protocol devised by Atkinson et al. was used with the following priorities: (1) the lag that the author focused on in the abstract or stated a priori, (2) the lag with the most statistical significance (positive or negative), and (3) the lag that showed the largest effect estimate (positive or negative) [15].

### Quality assessment

Due to the lack of validated scales for quality assessment of time-series and case-crossover studies, we adapted the quality assessment approach developed by Mustafic et al. [16]. Three components were assessed: (1) pneumonia diagnosis, where one point was given if the diagnosis of pneumonia was coded according to the International Classification of Diseases (ICD) or based on medical records; (2) the air pollutant measures, where one point was given if measurements were performed at least daily with less than 25% missing data; (3) adjustment for confounders, where one point was given if an adjustment for either long-term trends, seasonality, or temperature was made; a second point was given if an additional adjustment was performed either for humidity or day of the week; and a third point was given if a further adjustment was made for influenza epidemics or holidays. Studies that achieved maximum points for all three components were regarded as good quality, whereas those that achieved no points in any of the three components were regarded as low quality; the remaining were regarded as intermediate quality.

### Statistical analysis

We used % increase with 95% CIs as a measure of effect size. To pool the results, all estimates were standardized to an increase of 10 μg/m^3^ of PM_2.5_ and PM_10_ concentrations; 1 ppm of CO; 10 ppb of SO_2_, NO_2_, and O_3_. To transform the estimate, the following equation was used: odds ratio (OR)_standardized_ = OR^increment(10)/increment(original)^ [17]. As the authors of the original articles adjusted for the time-varying confounders, we extracted the adjusted values. Statistical significance was analyzed by Z-test and a *p*-value < 0.05 was considered statistically significant. Heterogeneity between studies was assessed by a chi square-based Q test and I^2^ test. A random-effects model (DerSimonian-Laird method) was applied to consider the heterogeneity within and between studies and to give a more conservative estimate of statistical confidence [18]. Publication bias was assessed using funnel plot and Begg’s test [19]. Sensitivity analysis using the leave-one-out method was performed to assess the stability of results. Subgroup analysis was also conducted per region. In addition, meta-analyses for combining the results with the same lag day were performed. All statistical analyses were performed using R software (version 3.6.0; R Foundation for Statistical Computing, Vienna, Austria). This review followed the Preferred Reporting Items for Systematic Reviews and Meta-analysis (PRISMA) guidelines [20].

## Results

**Fig. 1:**
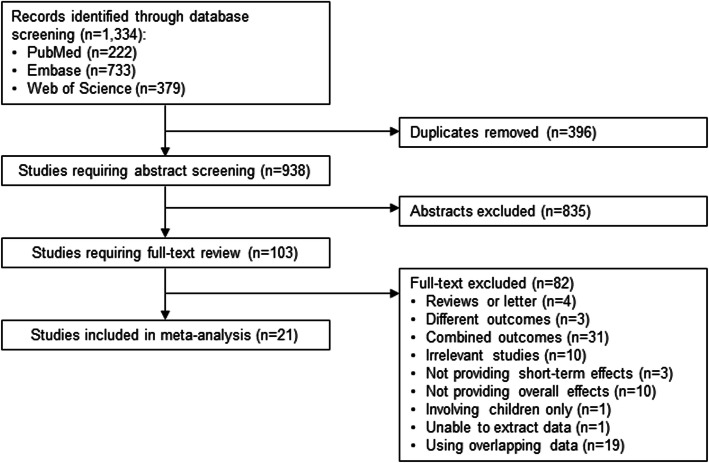
Flow diagram of study selection

The study selection process is summarized in Fig. 1. A total of 1334 records were identified from the three databases and 396 duplicates were excluded. After removing 835 studies during title and abstract screening, 103 were selected for full-text review, and 82 studies were excluded for the following reasons: reviews or letter (*n* = 4), different outcomes (*n* = 3), combined outcomes (*n* = 31), irrelevant studies (*n* = 10), not providing short-term effects (*n* = 3), not providing overall effects (*n* = 10), conducted in children (*n* = 1), unable to extract data (*n* = 1), and overlapping studies (*n* = 19). Finally, 21 studies were included for meta-analysis [9, 10, 21–39].

The characteristics of included studies are shown in Table 1. Of the 21 remaining studies, 14 were time-series studies and 7 were case-crossover studies. The United States of America was the country where the research was most conducted (*n* = 7), followed by China (*n* = 4). Most studies used the ICD code for pneumonia diagnosis; almost 60% of the studies examined hospital admission, while the rest examined ER visit. The number of cases in each study ranged from 325 to 4.2 million. In terms of study quality, about 43% of studies were regarded as good quality, while the rest were intermediate quality.

**Table 1:** Characteristics of included studies

Study ID	Location	Participants	Study period	Number of cases	Male (%)	Elderly (%)	Data source of outcome	Studied pollutants	Outcome level	Study design	Study quality
Chang 2017 [21]	Taipei, Taiwan	All ages	2012–2015	3729	NA	NA	Medical records (1 hospital)	PM_2.5_, SO_2_, NO_2_	Emergency room visit, ICD-9	Time-series	Intermediate
Cheng 2019 [22]	Kaohsiung, Taiwan	> 17 years	2007–2013	4015	63.5%	NA	Medical records (1 hospital)	PM_2.5_, PM_10_, SO_2_, NO_2_, O_3_	Emergency room visit, ICD-9	Case-crossover	Intermediate
Duan 2016 [9]	Shijiazhuang, China	≥18 years	2013	2253	53.4%	55%^a^	Medical records (7 hospitals)	PM_2.5_, PM_10_, SO_2_, NO_2_, CO, O_3_	Hospital admission, diagnosis	Case-crossover	Intermediate
Franck 2015 [23]	Santiago, Chile	All ages	2004–2007	44,430	NA	NA	FONASA and ISAPREs data	PM_2.5_, PM_10_, NO_2_, CO, O_3_	Hospital admission, ICD-10	Case-crossover	Intermediate
Halonen 2009 [24]	Helsinki, Finland	≥65 years	1998–2004	10,733	NA	100%	Statistics Finland	PM_2.5_	Hospital admission, ICD-10	Time-series	Good
Hinwood 2006 [25]	Perth, Australia	All ages	1992–1998	10,000 (estimated)	NA	NA	Medical records (all hospitals)	PM_2.5_, CO, O_3_	Unscheduled hospital admission, ICD-9	Case-crossover	Good
Kim 2012 [26]	Five counties in Denver metropolitan area, CO, USA	All ages	2003–2007	23,000 (estimated)	NA	44%^b^	Colorado Hospital Association data	PM_2.5_, SO_2_, NO_2_	Nonelective hospital admission, ICD-9	Time-series	Intermediate
Liu 2016 [27]	Greater Huston, TX, USA	All ages	2008–2013	1097	49.4%	12.6%	Blue Cross Blue Shield Texas claims data	PM_2.5_	Emergency hospital admission, ICD-9	Time-series	Intermediate
Malig 2013 [28]	CA, USA	All ages	2005–2008	70,967	NA	NA	2 databases maintained by the California OSHPD	PM_2.5_, PM_10_	Emergency room visit, ICD-9	Case-crossover	Intermediate
Medina-Ramon 2006 [10]	36 cities in USA	> 65 years	1986–1999	1,384,813	NA	100%	Medicare claims data	PM_10_, O_3_	Emergency hospital admission, ICD-9	Case-crossover	Intermediate
Pennington 2019 [29]	Atlanta, GA, USA	All ages	1998–2010	162,000 (estimated)	NA	NA	Medical records (41 hospitals)	PM_2.5_	Emergency room visit, ICD-9	Time-series	Good
Phosri 2019 [30]	Bangkok, Thailand	All ages	2006–2014	59,000 (estimated)	43.4%^c^	15.7%^c^	NHSO claims data	PM_10_, SO_2_, NO_2_, CO, O_3_	Hospital admission, ICD-10	Time-series	Good
Pothirat 2019 [31]	Chiang Mai, Thailand	All ages	2016–2017	325	NA	NA	Medical records (1 hospital)	PM_2.5_, PM_10_, SO_2_, NO_2_, CO, O_3_	Hospital admission, ICD-10	Time-series	Intermediate
Qiu 2014 [32]	Hong Kong	All ages	2011–2012	75,863	53.5%	74.5%	Hospital Authority Corporate Data Warehouse data	PM_2.5_	Emergency hospital admission, ICD-9	Time-series	Good
Rodopoulou 2015 [33]	Central AR, USA	≥15 years	2002–2012	2412	40.2%^c^	7.4%^c^	UAMS Enterprise Data Warehouse data	PM_2.5_, O_3_	Emergency room visit, ICD-9	Time-series	Good
Santus 2012 [34]	Milan, Italy	All ages	2007–2008	5689	54.9%	48.5%	Medical records (5 hospitals)	PM_2.5_, PM_10_, SO_2_, NO_2_, CO, O_3_	Emergency room visit, ICD-9	Case-crossover	Intermediate
Tao 2014 [35]	Lanzhou, China	All ages	2001–2005	4559	63.1%	18.5%	Medical records (4 hospitals)	PM_10_, SO_2_, NO_2_	Hospital admission, ICD-10	Time-series	Intermediate
Tasci 2018 [36]	Ankara, Turkey	> 65 years	2011–2015	2606	53.2%	100%	Medical records (1 hospital)	PM_2.5_, PM_10_, SO_2_, NO_2_, CO	Emergency room visit, diagnosis	Time-series	Intermediate
Tian 2019 [37]	184 cities in China	≥18 years	2014–2017	4.2 million	NA	NA	UEBMI claims data	PM_2.5_, PM_10_	Hospital admission, ICD-10	Time-series	Good
Tian 2020 [38]	184 cities in China	≥18 years	2014–2017	4.2 million	NA	NA	UEBMI claims data	O_3_	Hospital admission, ICD-10	Time-series	Good
Winquist 2012 [39]	St. Louis MSA, USA	All ages	2001–2007	98,000 (estimated)	NA	35.0%	Missouri Hospital Association data (28 hospitals)	PM_2.5_, O_3_	Emergency room visit, ICD-9	Time-series	Good

*NA* Not available, *MSA* Metropolitan statistical area, *FONASA* Fondo Nacional de Salud de Chile, *ISAREE* Instituciones de Salud Previsional, *OSHPD* Office of Statewide Health Planning and Development, *NHSO* National Health Security Office, *UAMS* University of Arkansas for Medical Sciences, *UEBMI* Urban Employee Basic Medical Insurance

^a^ ≥ 60 years

^b^All hospital admission

^c^Respiratory admission

**Fig. 2:**
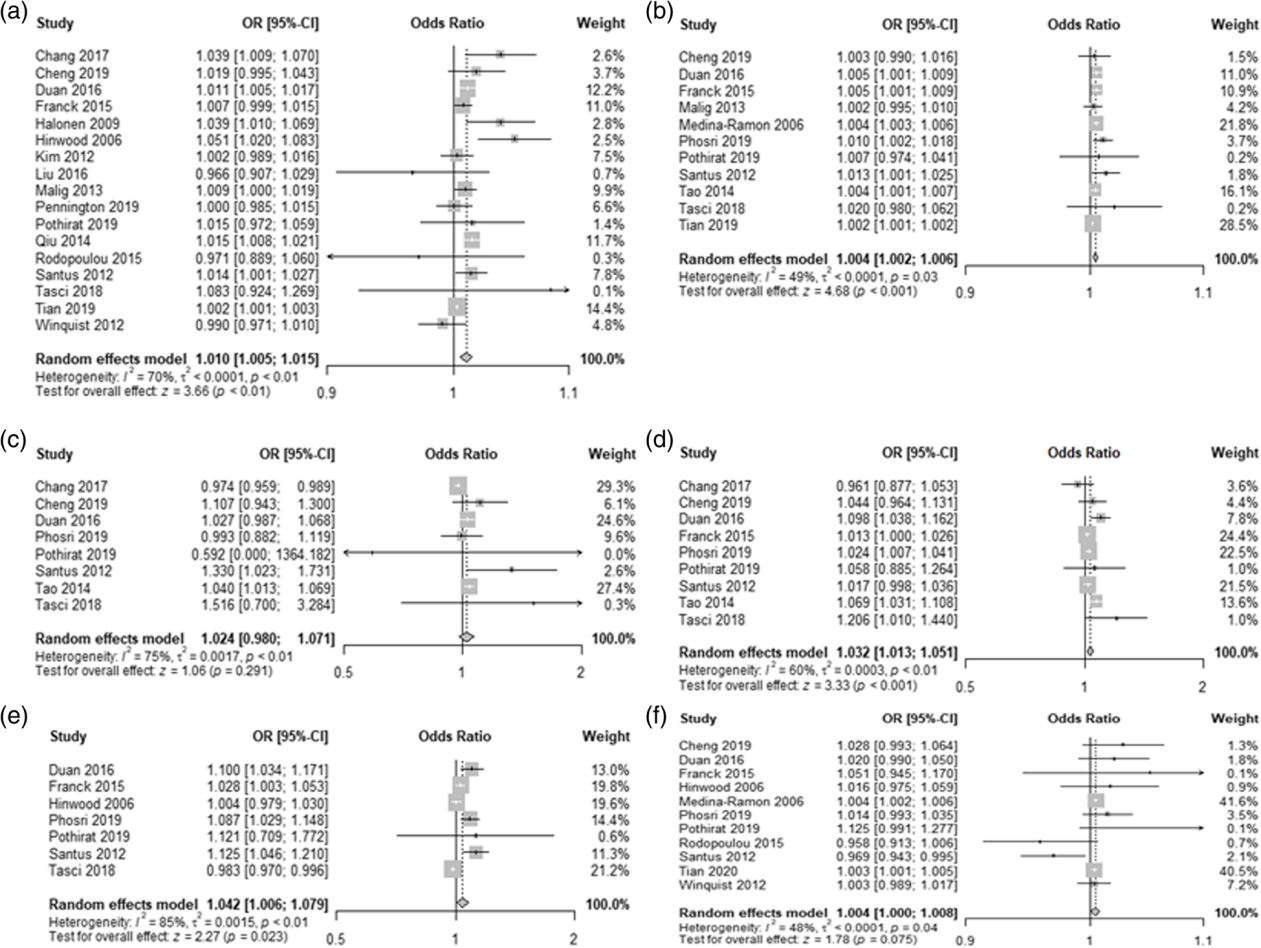
Forest plots of the association between air pollutants and hospital admission or emergency room visit for pneumonia. **a** PM_2.5_. **b** PM_10_. **c** SO_2_. **d** NO_2_. **e** CO. **f** O_3_

Among the air pollutants analyzed, PM_2.5_, PM_10_, NO_2_, and CO were associated with an increased risk of hospital admission or ER visit for pneumonia (Fig. 2). For PM_2.5_ and PM_10_, a 10 μg/m^3^ increase was associated with a 1.0% (95% CI: 0.5–1.5; I^2^ = 70%) and 0.4% (95% CI: 0.2–0.6; I^2^ = 49%) increase in hospital admission or ER visit for pneumonia, respectively. In addition, every 1 ppm increase of CO was associated with 4.2% (95% CI: 0.6–7.9; I^2^ = 85%) increase in hospital admission or ER visit for pneumonia. For every 10 ppb increase of NO_2_, SO_2_, and O_3_ increased pneumonia-specific hospital admission or ER visit by 3.2% (95% CI: 1.3–5.1; I^2^ = 60%), 2.4% (95% CI: -2.0-7.1; I^2^ = 75%), and 0.4% (95% CI: 0–0.8; I^2^ = 48%), respectively.

The publication bias was assessed using the funnel plot and Begg’s test (See Supplementary Figure 1, Additional file 1) and no evidence of publication bias was found (*P* > 0.05 for all analyses). Sensitivity analysis was performed by sequentially excluding each study. With the exception of CO, all pollutants, which obtained statistical significance in the main analyses, showed similar results, indicating that no individual study significantly affected the pooled results. The ranges of increase were 0.9–1.1% for PM_2.5_, 0.4–0.5% for PM_10_, and 2.4–4.0% for NO_2_. When excluding the three largest studies, which had more than 1 million cases [10, 37, 38], the pooled % increase of hospital admission or ER visit per 10 μg/m^3^ increase in PM_2.5_ and PM_10_ and 10 ppb in O_3_ was 1.1% (95% CI: 0.6–1.6; I^2^ = 46.5%), 0.5% (95% CI: 0.3–0.7; I^2^ = 0%), and 5.5% (95% CI: -1.1-2.2; I^2^ = 56%), respectively.

As there were six and five studies for PM_2.5_ conducted in North America and East Asia, respectively, we performed a subgroup analysis. Analysis on East Asia showed that every 10 μg/m^3^ increase of PM_2.5_ was associated with a 1.2% (95% CI: 0.3–2.0) increase in hospital admission or ER visit for pneumonia, whereas the effect estimate for North America was considerably smaller than that for East Asia; yet the confidence intervals still exhibited considerable overlap (0.3, 95% CI: − 0.4-1.0). For PM_10_, East Asia and North America showed a 0.3% (95% CI: 0.1–0.4) and 0.4% (95% CI: 0.3–0.6) increase for every 10 μg/m^3^ increase, respectively; for O_3_, the corresponding values were 0.9% (95% CI: − 0.5-2.4) and 0.2% (95% CI: − 0.8-1.2). In terms of SO_2_, NO_2_ and CO, there were no available studies conducted on North America.

When combining results with the same lag day, PM_2.5_ and PM_10_ showed significant associations with pneumonia-specific hospital admission or ER visit for all lag days (lag 0 to lag 5) and the largest association was observed for lag 3 and lag 5, respectively (See Supplementary Table 2, Additional file 1). The % increase range per 10 μg/m^3^ increase for PM_2.5_ at lag 3 and PM_10_ at lag 5 was 1.0% (95% CI: 0.4–1.6) and 0.4% (95% CI: 0.2–0.6), respectively. For NO_2_ and CO, the largest association was observed for lag 2 (% increase: 2.1, 95% CI: 0.1–4.2) and lag 5 (% increase: 29.8, 95% CI: 0.8–67.2), respectively. On the contrary, SO_2_ and O_3_ levels did not show significant associations for all lag days (lag 0 to lag5), and the % increase range for SO_2_ and O_3_ was − 0.3 to 1.9 and − 1.0 to 0.7, respectively.

## Discussion

In this meta-analysis, we demonstrated a significant association between air pollutants (PM_2.5_, PM_10_, NO_2_, and CO) and hospital admission or ER visit for pneumonia, although no such association was identified regarding SO_2_ and O_3._ Except for CO, the sensitivity analyses yielded similar results, demonstrating the robustness of the results. In a subgroup analysis by region, PM_2.5_ increased hospital admission or ER visit for pneumonia in East Asia but not in North America.

PM has been associated with cardiovascular hospitalization in several meta-analysis studies [8, 40]. PM_2.5_ was reported to even increase cardiovascular mortality by approximately 0.4 to 1.0% for every 10 μg/m^3^ [41]. Associations have also been reported between PM and other respiratory diseases, including asthma and COPD [13, 14]. It was shown that asthma- and COPD-specific hospital admission increased by almost 2% for every 10 μg/m^3^ increase in PM_2.5_. Our findings are also consistent with a recent meta-analysis on short-term PM and pneumonia in children, which used similar meta-analytic methods, including time-series and case-crossover studies, and according to Nhung et al.*,* a 10 μg/m^3^ increment of PM_2.5_ and PM_10_ was associated with a 1.8 and 1.5% increase in pneumonia hospital admission, respectively [42]. The higher estimates in children compared to our results may be attributed to their increased inhalation per body weight and immature immune systems, rendering them more susceptible to infections [43, 44].

Several studies have suggested that PM is related to inflammation. According to Gordon et al.*,* PM_2.5_ is linked to an increase in pro-inflammatory cytokines (interleukin-1, interleukin-6, and tumor necrosis factor-α) and Th1-type cytokines (interleukin-12 and interferon-γ) [45]. Elevated levels of white blood cells, C-reactive protein, and von Willebrand factor, which are involved in systemic inflammation, have also been observed after PM exposure [46, 47].

There are two possible mechanisms that mainly account for the increased risk of pneumonia induced by PM: altered immunity and oxidative stress, both of which are closely linked to inflammation. The respiratory system possesses multiple tiers of immunity to defend against harmful airborne particles and microorganisms [48]. However, despite its complex protective mechanisms, several studies have shown that PM_2.5_ exposure can damage the mucociliary system [49], suppress alveolar macrophage uptake [50], and impair microbial clearance [51], whereby it can enhance pneumococcal adherence to airway epithelial cells [52]. Although PM_2.5_, which can be inhaled more deeply into the lungs, is considered more harmful to health than PM_10_, the latter can also cause pneumonia in a similar manner [41].

Oxidative stress is another factor believed to play an important role in the pathogenesis of PM-induced pneumonia. Several studies have shown that acute exposure to PM triggers pulmonary oxidative stress. PM can directly generate reactive oxygen species on the surface, alter mitochondrial function, dysregulate antioxidant enzymes (e.g., superoxide dismutase), increase other oxidases in the lungs (e.g., inducible nitric oxide synthase), and activate metabolic enzyme activity including cytochrome P450s and glutathione S-transferase [53, 54]. These responses can cause pulmonary oxidative damage, which induces an inflammatory process in the lungs [55].

Along with PM, NO_2_, a free-radical gaseous component of indoor and outdoor air pollution, was associated with increased risk of pneumonia hospital admission or ER visit in this study. As NO_2_ is linked to nitrosative stress in the lungs, it can lead to airway injury [56]. Furthermore, animal studies have shown that exposure to NO_2_ results in increased susceptibility to both bacterial and viral infections [57, 58], which explains the positive association between NO_2_ and pneumonia.

CO was another air pollutant associated with pneumonia. According to Ghio et al.*,* CO can trigger proinflammatory responses in the airways [59]. In addition, previous studies have reported the association between CO and other respiratory diseases, including asthma and COPD [13, 60, 61]. However, in the sensitivity analysis, the association did not remain significant, especially when studies of Duan et al. [9], Phosri et al. [30], and Santus et al. [34] were excluded separately, possibly because the studies showed strong associations between CO and pneumonia in addition to having considerable weight. Therefore, caution is required to interpret the association between CO and pneumonia-specific hospital admission or ER visit.

Subgroup analysis by region showed that an increase of PM_2.5_ was associated with risk of hospital admission or ER visit for pneumonia in East Asia but not in North America. The two regions are known to have remarkably different concentrations of PM_2.5_ with East Asia generally having a higher exposure to PM_2.5_ and its consequent health burden than North America [62]. In addition, regional differences could be accounted for by the variability in composition and toxicity of air pollutants and/or variations in population susceptibility. As there have been insufficient studies investigating the direct effects of PM_2.5_ on pneumonia in different countries, further research is required on the effects of air pollutants between different regions.

We performed meta-regression with the confounder-adjusting method, which is assessed on a 0 to 3 scale by the third component of quality assessment (described in the method section). Except for PM_10_, no association between air pollutants and pneumonia was affected by the confounder-adjusting method. Although meta-regression analysis showed that the confounder-adjusting method affected the pooled estimates for PM_10_, most studies on PM_10_ yielded the same score (2 point); therefore, the result was not confirmative.

This meta-analysis has some limitations that should be considered when interpreting the results. First, included studies used the air pollutant levels obtained from monitoring stations rather than personal exposures. Second, considerable heterogeneity was observed. Third, due to the lack of information from individual studies, some potential factors, which could affect the risk of pneumonia-specific hospital admission or ER visit (e.g., patients’ age or comorbidities), could not be adjusted.

## Conclusions

To our knowledge, this is the first systematic review and meta-analysis to evaluate the acute effects of air pollutants on hospital admission or ER visit for pneumonia. By combining the inconsistent findings of several studies, this study revealed the associations between short-term exposure of air pollutants and pneumonia-specific hospital admission or ER visit, especially for PM and NO_2_. Based on the results, stricter intervention policies regarding air pollution and programs for protecting human respiratory health should be implemented.

## Supplementary Information

**Additional file 1: Supplementary Table 1.** Search strategy. **Supplementary Table 2.** Meta-analyses of association between air pollutants and hospital admission or emergency room visit for pneumonia in the same lag day. **Supplementary Figure 1.** Funnel plot of association between air pollutants and hospital admission or emergency room visit for pneumonia. a. Funnel plot of association between PM_2.5_ and hospital admission or emergency room visit for pneumonia. b. Funnel plot of association between PM_10_ and hospital admission or emergency room visit for pneumonia. c. Funnel plot of association between SO_2_ and hospital admission or emergency room visit for pneumonia. *D.* funnel plot of association between NO_2_ and hospital admission or emergency room visit for pneumonia. e. Funnel plot of association between CO and hospital admission or emergency room visit for pneumonia. f. Funnel plot of association between O_3_ and hospital admission or emergency room visit for pneumonia. **Supplementary Figure 2.** Subgroup analysis by region for the association between air pollutants and hospital admission or emergency room visit for pneumonia. a. Subgroup analysis by region for the association between PM_2.5_ and hospital admission or emergency room visit for pneumonia. b. Subgroup analysis by region for the association between PM_10_ and hospital admission or emergency room visit for pneumonia. c. Subgroup analysis by region for the association between O_3_ and hospital admission or emergency room visit for pneumonia. **Supplementary Figure 3.** Subgroup analysis by study design for the association between air pollutants and hospital admission or emergency room visit for pneumonia. a. Subgroup analysis by study design for the association between PM_2.5_ and hospital admission or emergency room visit for pneumonia. b. Subgroup analysis by study design for the association between PM_10_ and hospital admission or emergency room visit for pneumonia. c. Subgroup analysis by study design for the association between SO_2_ and hospital admission or emergency room visit for pneumonia. d. Subgroup analysis by study design for the association between NO_2_ and hospital admission or emergency room visit for pneumonia. e. Subgroup analysis by study design for the association between CO and hospital admission or emergency room visit for pneumonia. f. Subgroup analysis by study design for the association between O_3_ and hospital admission or emergency room visit for pneumonia. **Supplementary Figure 4.** Subgroup analysis by study quality for the association between air pollutants and hospital admission or emergency room visit for pneumonia. a. Subgroup analysis by study quality for the association between PM_2.5_ and hospital admission or emergency room visit for pneumonia. b. Subgroup analysis by study quality for the association between PM_10_ and hospital admission or emergency room visit for pneumonia. c. Subgroup analysis by study quality for the association between SO_2_ and hospital admission or emergency room visit for pneumonia. d. Subgroup analysis by study quality for the association between NO_2_ and hospital admission or emergency room visit for pneumonia. e. Subgroup analysis by study quality for the association between CO and hospital admission or emergency room visit for pneumonia. f. Subgroup analysis by study quality for the association between O_3_ and hospital admission or emergency room visit for pneumonia.

## Data Availability

The datasets used and/or analysed during the current study are available from the corresponding author on reasonable request.

## Abbreviations

CIs: Confidence intervals
COPD: Chronic obstructive pulmonary disease
ER: Emergency room
ICD: International Classification of Diseases
ORs: Odds ratios
PM: Particulate matter
PRISMA: Preferred Reporting Items for Systematic Reviews and Meta-analysis
WHO: World Health Organization
