# Supplementary material for: Short-term exposure to air pollution and hospital admission for pneumonia: a systematic review and meta-analysis
Source: Environ Health. 2021 Jan 7;20:6. doi: 10.1186/s12940-020-00687-7 (PMC7792212; doi:10.1186/s12940-020-00687-7)

**Supplementary Table 1.** Search strategy

| No | Search term | Pubmed | Embase | Web of Science |
| --- | --- | --- | --- | --- |
| #1 | (air pollutant*) or (air pollution*) or (air qualit*) | 132,727 | 143,025 | 177,045 |
| #2 | (particulate matter*) or (fine particulate*) or (fine particle*) or (coarse particulate*) or (coarse particle*) | 31,087 | 119,971 | 72,841 |
| #3 | (nitrogen dioxide) or (nitrogen oxide*) | 24,533 | 83,213 | 56,671 |
| #4 | (ozone) | 23,087 | 62,409 | 36,238 |
| #5 | (sulfur dioxide) or (sulphur dioxide) or (sulfurous anhydride) or (sulphurous anhydride) | 9,312 | 18,382 | 19,818 |
| #6 | (carbon monoxide) | 35,866 | 59,041 | 51,891 |
| #7 | #1 or #2 or #3 or #4 or #5 or #6 | 204,544 | 410,633 | 329,593 |
| #8 | (pneumonia*) | 204,857 | 149,098 | 395,526 |
| #9 | (lower respiratory infection*) or (lower respiratory tract infection*) | 7,605 | 23,018 | 42,743 |
| #10 | #8 or #9 | 209,608 | 165,457 | 424,570 |
| #11 | (hospitalization*) or (hospitalisation*) or (admission*) or (hospital visit*) or (emergency visit*) | 403,428 | 307,679 | 997,149 |
| #12 | #10 and #11 | 19,578 | 18,105 | 58,417 |
| #13 | #7 and #12 | 222 | 379 | 733 |

**Supplementary Table 2.** Meta-analyses of association between air pollutants and hospital admission or emergency room visit for pneumonia in the same lag day.

| Components | Lag day | Number of studies | Odds ratio | Lower 95% CI | Higher 95% CI | I^2^ |
| --- | --- | --- | --- | --- | --- | --- |
| PM_2.5_ | Lag 0 | 14 | **1.0045** | **1.0020** | **1.0070** | 12.9% |
|  | Lag 1 | 13 | **1.0044** | **1.0002** | **1.0087** | 53.0% |
|  | Lag 2 | 13 | **1.0043** | **1.0015** | **1.0072** | 19.6% |
|  | Lag 3 | 11 | **1.0100** | **1.0042** | **1.0159** | 43.9% |
|  | Lag 4 | 8 | **1.0070** | **1.0011** | **1.0129** | 56.7% |
|  | Lag 5 | 7 | **1.0073** | **1.0018** | **1.0129** | 46.3% |
| PM_10_ | Lag 0 | 9 | **1.0026** | **1.0014** | **1.0039** | 20.9% |
|  | Lag 1 | 9 | **1.0021** | **1.0007** | **1.0035** | 24.8% |
|  | Lag 2 | 8 | **1.0021** | **1.0012** | **1.003** | 0.0% |
|  | Lag 3 | 6 | **1.0040** | **1.0014** | **1.0067** | 0.0% |
|  | Lag 4 | 5 | **1.0029** | **1.0002** | **1.0056** | 0.0% |
|  | Lag 5 | 5 | **1.0043** | **1.0024** | **1.0063** | 0.0% |
| SO_2_ | Lag 0 | 6 | 0.9970 | 0.9612 | 1.0341 | 34.0% |
|  | Lag 1 | 6 | 1.0175 | 0.9877 | 1.0483 | 35.2% |
|  | Lag 2 | 7 | 1.0191 | 0.9926 | 1.0464 | 43.0% |
|  | Lag 3 | 6 | 1.0135 | 0.9859 | 1.0418 | 35.1% |
|  | Lag 4 | 5 | 1.0050 | 0.9916 | 1.0184 | 0.0% |
|  | Lag 5 | 4 | 1.0013 | 0.9754 | 1.0279 | 22.4% |
| NO_2_ | Lag 0 | 7 | **1.0150** | **1.0031** | **1.0271** | 5.0% |
|  | Lag 1 | 7 | 1.0091 | 0.9951 | 1.0232 | 16.0% |
|  | Lag 2 | 8 | **1.0209** | **1.0006** | **1.0416** | 52.7% |
|  | Lag 3 | 7 | 1.0111 | 0.9973 | 1.0251 | 18.1% |
|  | Lag 4 | 6 | 1.0132 | 0.9917 | 1.0352 | 52.3% |
|  | Lag 5 | 5 | 1.0213 | 0.9997 | 1.0434 | 48.9% |
| CO | Lag 0 | 6 | 1.3106 | 0.9315 | 1.8441 | 82.0% |
|  | Lag 1 | 6 | **1.2037** | **1.0197** | **1.4209** | 30.5% |
|  | Lag 2 | 6 | 1.1309 | 0.9266 | 1.3802 | 47.9% |
|  | Lag 3 | 6 | 1.1848 | 0.9536 | 1.472 | 55.3% |
|  | Lag 4 | 5 | 1.1668 | 0.8445 | 1.6119 | 70.5% |
|  | Lag 5 | 4 | **1.2977** | **1.0075** | **1.6715** | 9.2% |
| O_3_ | Lag 0 | 8 | 1.0072 | 0.9977 | 1.0168 | 90.1% |
|  | Lag 1 | 8 | 1.0019 | 0.9979 | 1.0059 | 48.1% |
|  | Lag 2 | 6 | 0.9992 | 0.9818 | 1.0169 | 9.6% |
|  | Lag 3 | 6 | 0.9982 | 0.9827 | 1.0140 | 0.0% |
|  | Lag 4 | 4 | 0.9898 | 0.9693 | 1.0108 | 21.2% |
|  | Lag 5 | 4 | 0.9939 | 0.9640 | 1.0248 | 51.0% |

Values in bold are statistically significant.

**Supplementary Figure 1.** Funnel plot of association between air pollutants and hospital admission or emergency room visit for pneumonia.

Supplementary Figure 1a. Funnel plot of association between PM_2.5_ and hospital admission or emergency room visit for pneumonia.


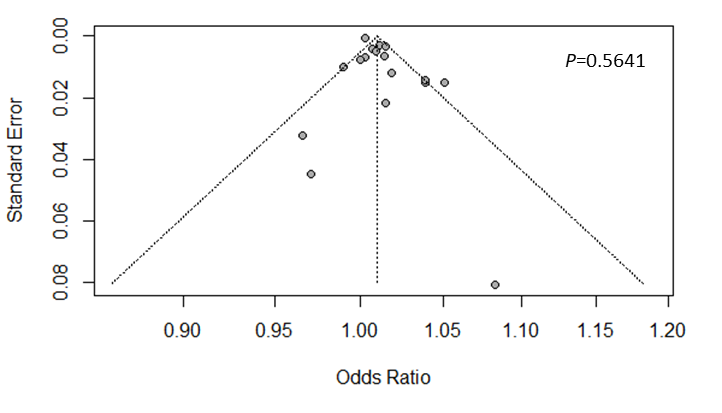


Supplementary Figure 1b. Funnel plot of association between PM_10_ and hospital admission or emergency room visit for pneumonia.


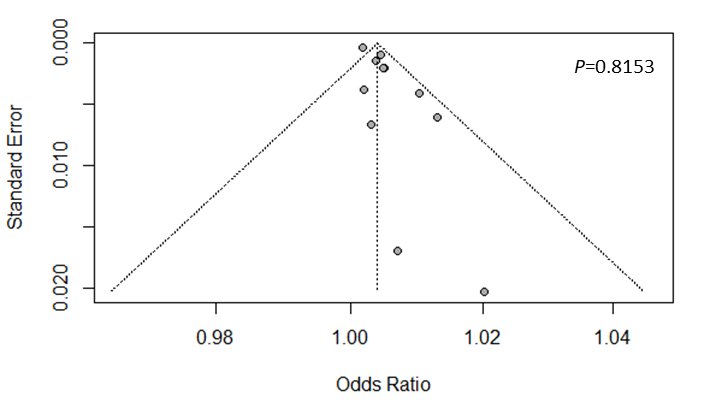


Supplementary Figure 1c. Funnel plot of association between SO_2_ and hospital admission or emergency room visit for pneumonia.


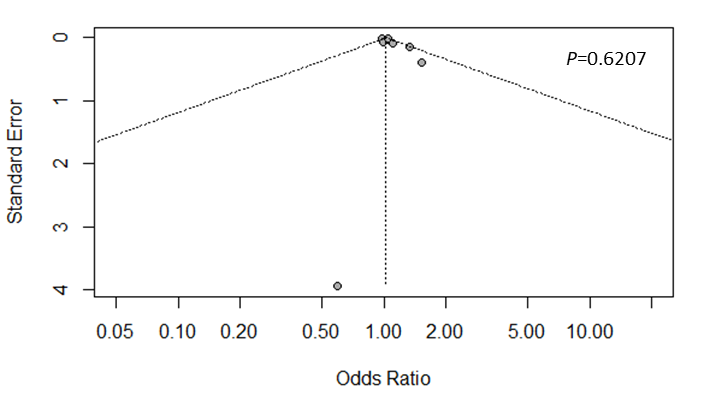


Supplementary Figure 1d. Funnel plot of association between NO_2_ and hospital admission or emergency room visit for pneumonia.


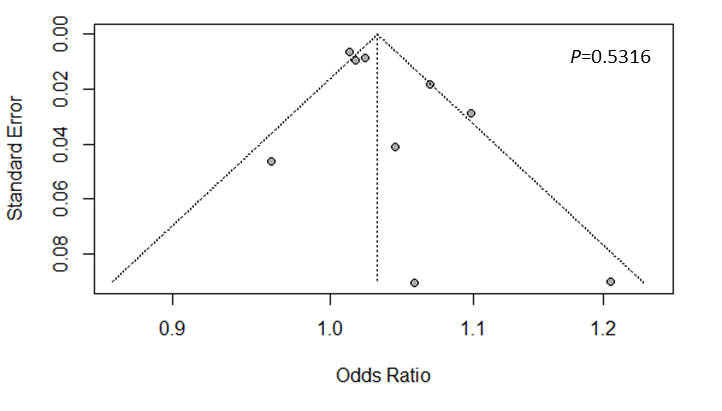


Supplementary Figure 1e. Funnel plot of association between CO and hospital admission or emergency room visit for pneumonia.


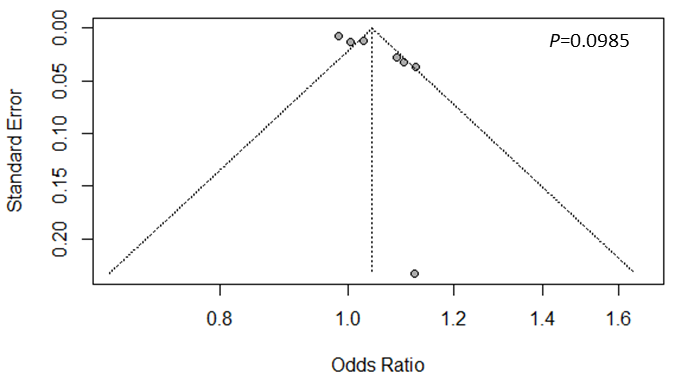


Supplementary Figure 1f. Funnel plot of association between O_3_ and hospital admission or emergency room visit for pneumonia.


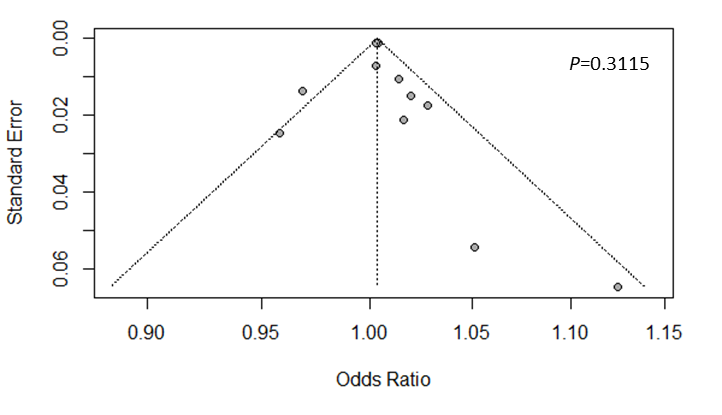


**Supplementary Figure 2.** Subgroup analysis by region for the association between air pollutants and hospital admission or emergency room visit for pneumonia.

Supplementary Figure 2a. Subgroup analysis by region for the association between PM_2.5_ and hospital admission or emergency room visit for pneumonia.


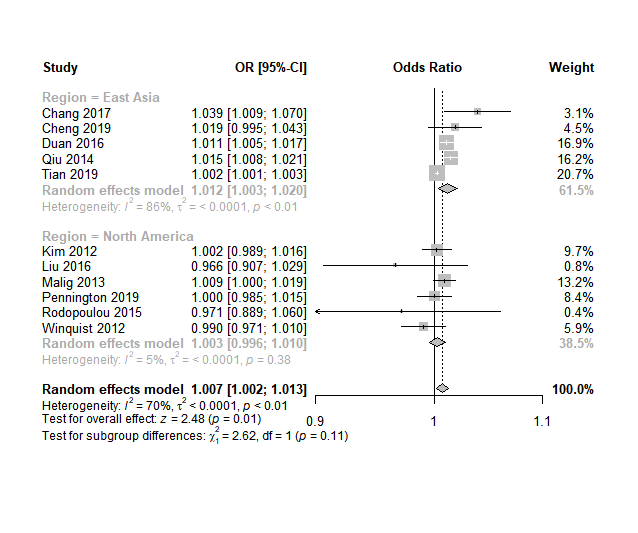


Supplementary Figure 2b. Subgroup analysis by region for the association between PM_10_ and hospital admission or emergency room visit for pneumonia.


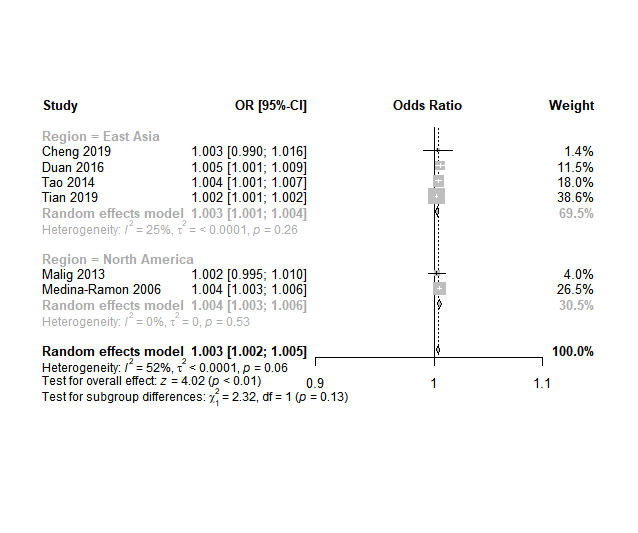


Supplementary Figure 2c. Subgroup analysis by region for the association between O_3_ and hospital admission or emergency room visit for pneumonia.


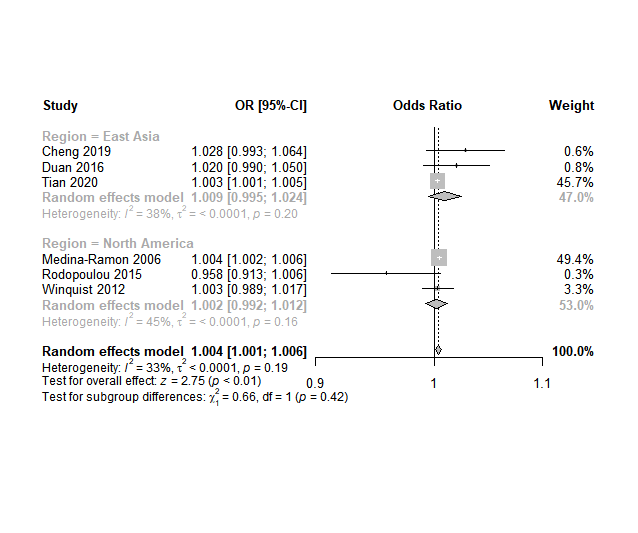


**Supplementary Figure 3.** Subgroup analysis by study design for the association between air pollutants and hospital admission or emergency room visit for pneumonia.

Supplementary Figure 3a. Subgroup analysis by study design for the association between PM_2.5_ and hospital admission or emergency room visit for pneumonia.


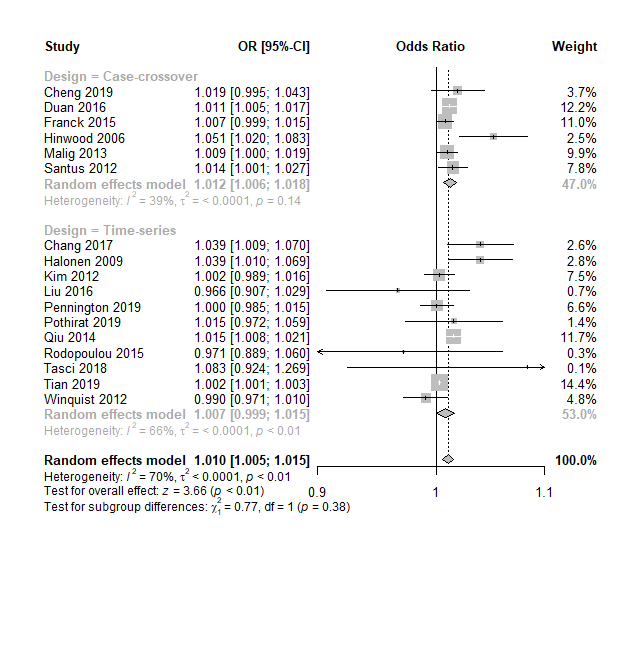


Supplementary Figure 3b. Subgroup analysis by study design for the association between PM_10_ and hospital admission or emergency room visit for pneumonia.


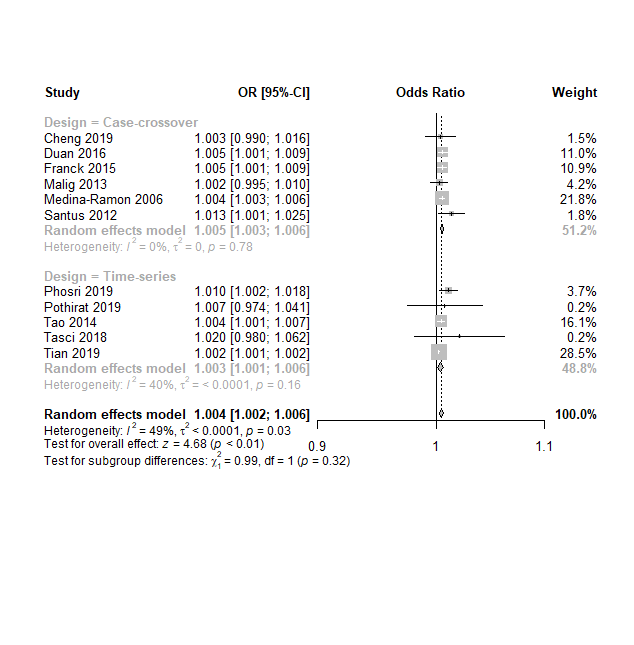


Supplementary Figure 3c. Subgroup analysis by study design for the association between SO_2_ and hospital admission or emergency room visit for pneumonia.


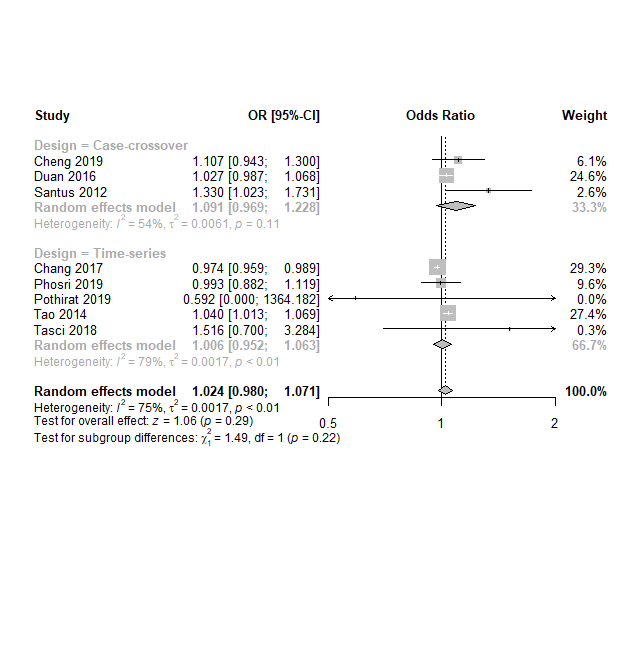


Supplementary Figure 3d. Subgroup analysis by study design for the association between NO_2_ and hospital admission or emergency room visit for pneumonia.


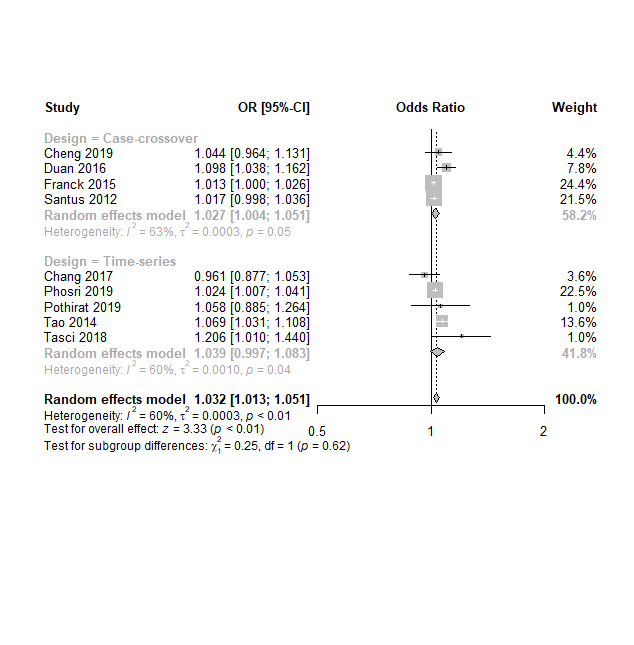


Supplementary Figure 3e. Subgroup analysis by study design for the association between CO and hospital admission or emergency room visit for pneumonia.


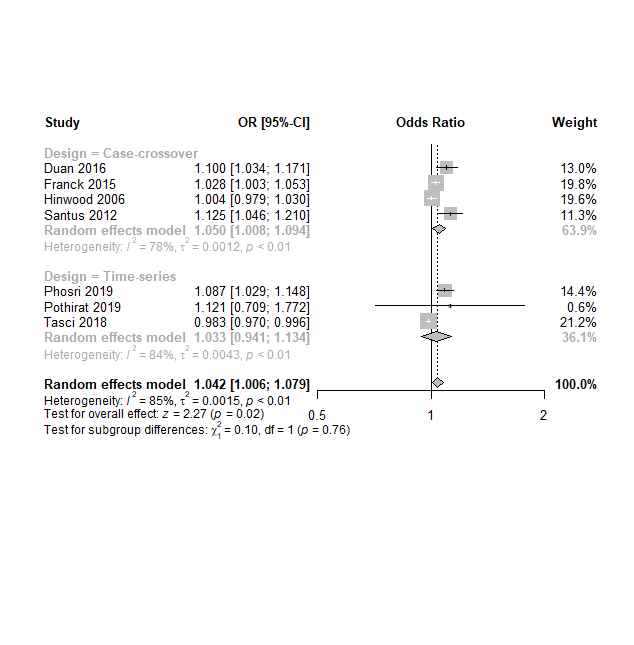


Supplementary Figure 3f. Subgroup analysis by study design for the association between O_3_ and hospital admission or emergency room visit for pneumonia.


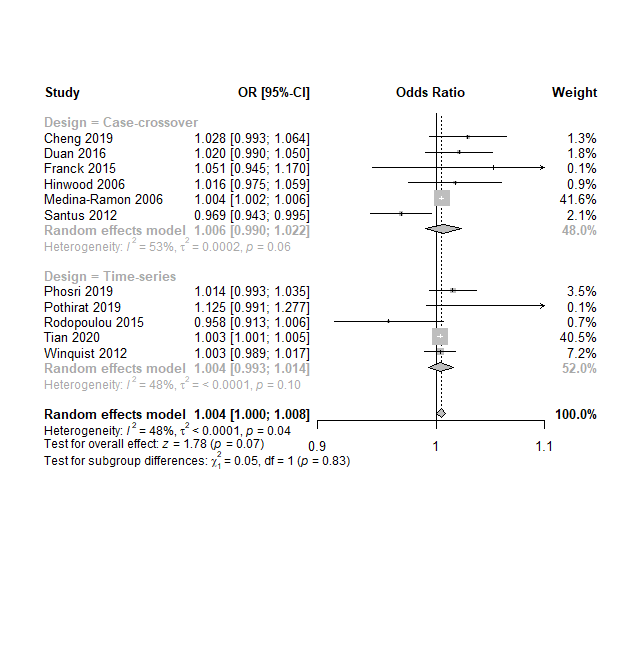


**Supplementary Figure 4.** Subgroup analysis by study quality for the association between air pollutants and hospital admission or emergency room visit for pneumonia.

Supplementary Figure 4a. Subgroup analysis by study quality for the association between PM_2.5_ and hospital admission or emergency room visit for pneumonia.


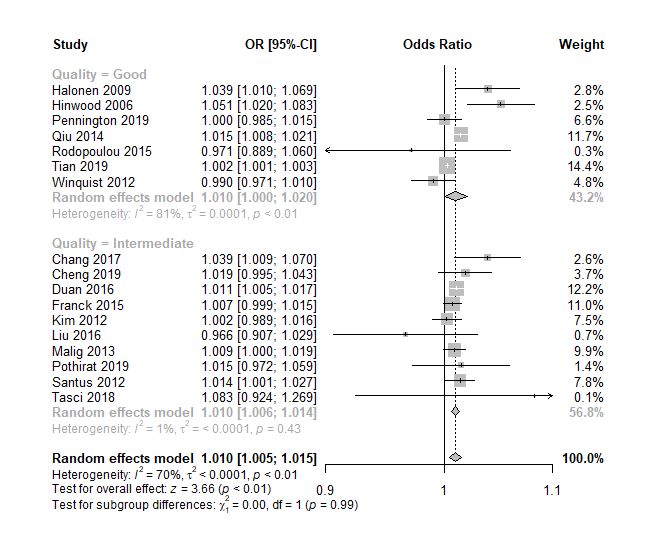


Supplementary Figure 4b. Subgroup analysis by study quality for the association between PM_10_ and hospital admission or emergency room visit for pneumonia.


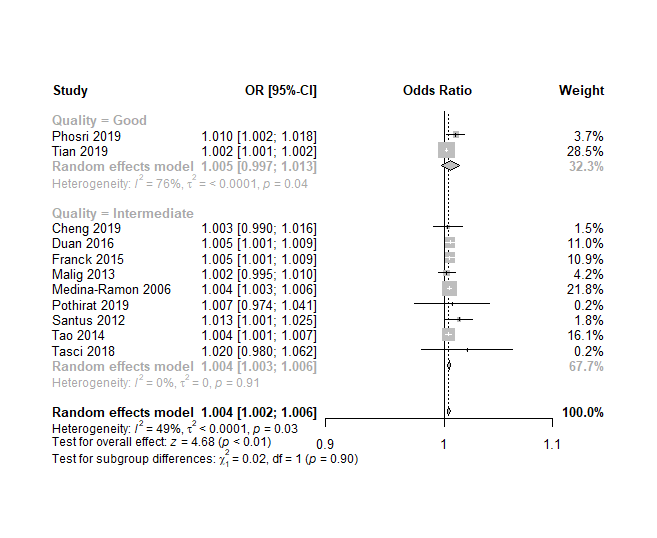


Supplementary Figure 4c. Subgroup analysis by study quality for the association between SO_2_ and hospital admission or emergency room visit for pneumonia.


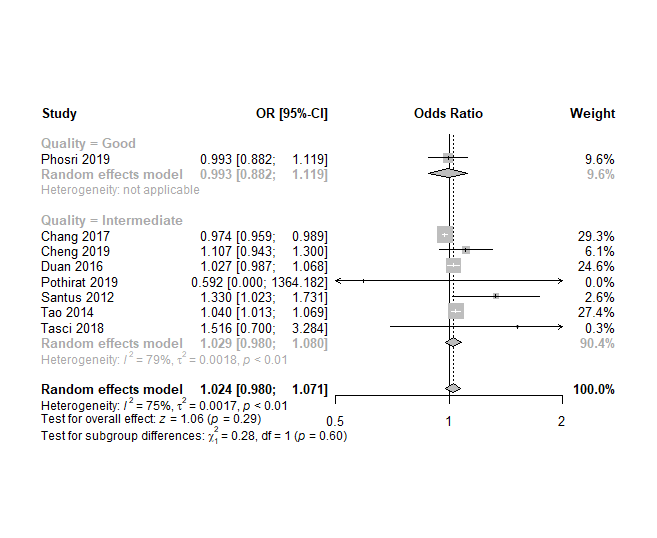


Supplementary Figure 4d. Subgroup analysis by study quality for the association between NO_2_ and hospital admission or emergency room visit for pneumonia.


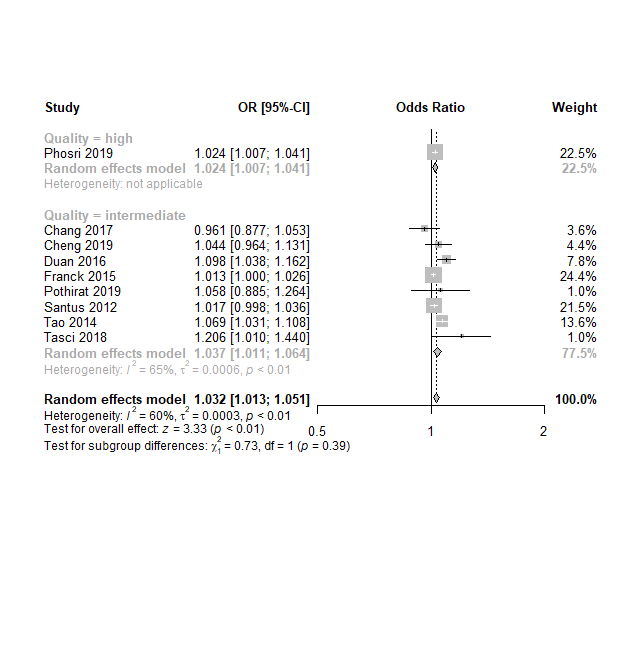


Supplementary Figure 4e. Subgroup analysis by study quality for the association between CO and hospital admission or emergency room visit for pneumonia.


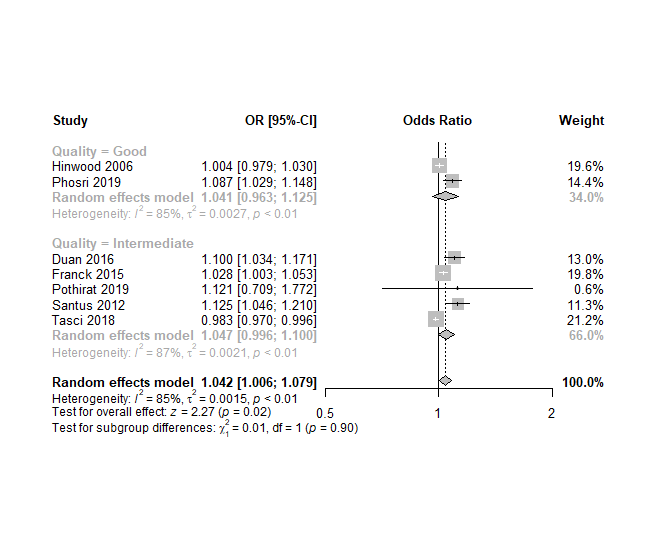


Supplementary Figure 4f. Subgroup analysis by study quality for the association between O_3_ and hospital admission or emergency room visit for pneumonia.


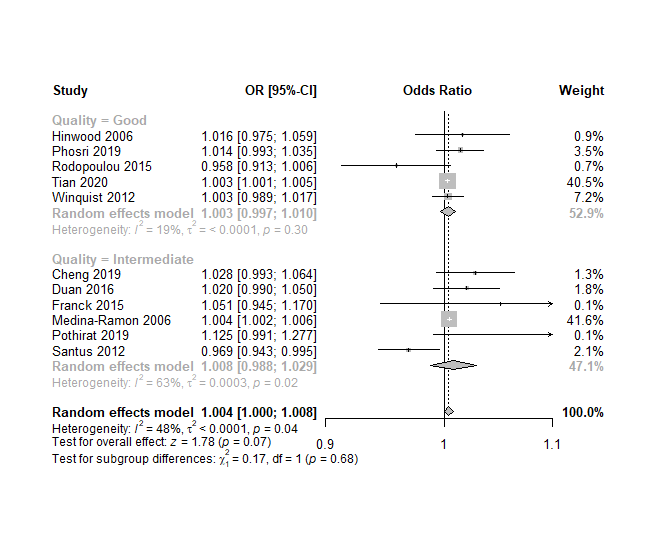

Supplement: Supplementary file 1 — Additional file 1: Supplementary Table 1. Search strategy. Supplementary Table 2. Meta-analyses of association between air pollutants and hospital admission or emergency room visit for pneumonia in the same lag day. Supplementary Figure 1. Funnel plot of association between air pollutants and hospital admission or emergency room visit for pneumonia. a. Funnel plot of association between PM2.5 and hospital admission or emergency room visit for pneumonia. b. Funnel plot of association between PM10 and hospital admission or emergency room visit for pneumonia. c. Funnel plot of association between SO2 and hospital admission or emergency room visit for pneumonia. D. funnel plot of association between NO2 and hospital admission or emergency room visit for pneumonia. e. Funnel plot of association between CO and hospital admission or emergency room visit for pneumonia. f. Funnel plot of association between O3 and hospital admission or emergency room visit for pneumonia. Supplementary Figure 2. Subgroup analysis by region for the association between air pollutants and hospital admission or emergency room visit for pneumonia. a. Subgroup analysis by region for the association between PM2.5 and hospital admission or emergency room visit for pneumonia. b. Subgroup analysis by region for the association between PM10 and hospital admission or emergency room visit for pneumonia. c. Subgroup analysis by region for the association between O3 and hospital admission or emergency room visit for pneumonia. Supplementary Figure 3. Subgroup analysis by study design for the association between air pollutants and hospital admission or emergency room visit for pneumonia. a. Subgroup analysis by study design for the association between PM2.5 and hospital admission or emergency room visit for pneumonia. b. Subgroup analysis by study design for the association between PM10 and hospital admission or emergency room visit for pneumonia. c. Subgroup analysis by study design for the association betw [file 12940_2020_687_MOESM1_ESM.docx]
